# A new cave-dwelling species of *Deuteraphorura* from northern Italy (Collembola, Onychiuridae)

**DOI:** 10.3897/zookeys.739.20923

**Published:** 2018-02-22

**Authors:** Pietro Paolo Fanciulli, Roberto Fabbri, Antonio Carapelli

**Affiliations:** 1 Department of Life Sciences, University of Siena, via Aldo Moro 2, 53100 Siena, Italy; 2 Museo Civico delle Cappuccine, Sezione Naturalistica “Pietro Bubani”, via Vittorio Veneto 1, 48012 Bagnacavallo (RA), Italy

**Keywords:** Chaetotaxy, Onychiuridae, springtails, taxonomy

## Abstract

A new cave-dwelling species, *Deuteraphorura pieroluccii* **sp. n.**, is described from northern Italy. The size of adult specimens, number, and distribution of dorsal and ventral pseudocelli, and number of compound vesicles of the post antennal organ were used to distinguish it among other congeneric species.

## Introduction

Several species of *Deuteraphorura* have been recently described, or re-described (Gruia and Popa 2005, Dallai and Fanciulli 2009, Fanciulli et al. 2010, Jordana et al. 2012, Weiner and Fiera 2014, Arbea and Park 2015, Parimuchová and Kováč 2016, Arbea 2017), updating the total number of the species of the genus to 83 (Bellinger et al. 1996–2017). The defining characters of the genus *Deuteraphorura* appear to be well-established due to the revision carried out by Weiner (1996) and Pomorski (1998), and to the introduction of some updated systematic keys (Jordana et al. 2012; Weiner and Fiera 2014). The Italian fauna of *Deuteraphorura* includes 16 species both from caves and open habitats (Fanciulli et al. 2010), with most of them being endemic along the Italian peninsula. In the present paper, a new cave-dwelling species of *Deuteraphorura* is described from northern Italy; furthermore, an updated key of the species recorded on the Italian peninsula is proposed. The new species was already reported as *Deuteraphorura* sp. in Fabbri and Poletti (2015).

## Materials and methods

Specimens of *Deuteraphorura pieroluccii* sp. n. were collected by hand inside the caves “Abisso Luigi Fantini” and “Buco del Noce” located very close to each other in the Messinian gypsum outcrops of the Vena del Gesso Romagnola (Brisighella, Ravenna Province, northern Italy) and preserved in 75% alcohol, until their preparation. The two caves both have small entrances. Abisso Luigi Fantini Cave (cave code ER RA 121; 44°13'23.08"N; 11°44'31.84"E) has an entrance at 426 m asl, spatial development of 1500 m and elevation difference of 117 m. Buco del Noce Cave (cave code ER RA 107; 44°13'34.68"N, 11°45'39.61"E) has entrance at 233 m asl, spatial development of 384 m and elevation difference of 43 m (Gruppo Speleologico Faentino & Speleo GAM Mezzano 2015). The specimens were subsequently cleared with lactic acid and mounted on slides with Gisin and Marc Andrè II solutions. Observations were performed with a Leitz Laborlux S microscope equipped with a camera lucida. In the description, the nomenclature proposed by Weiner (1996), Pomorski (1998), Jordana et al. (1997, 2012), and Fjellberg (1999) were considered in addition to the latest descriptions of Fanciulli et al. (2010) and Weiner and Fiera (2014). Pseudocellar formulae correspond to the number of pseudocelli per half-tergum/ half-sternum.

### Abbreviation used in the text

**Th** thoracic segment,

**Abd** abdominal segment,

**Ant** antennal segment,

**AOIII** sensory organ of antennal III segment,

**ms** microsensillum,

**PAO** postantennal organ,

**pso** pseudocellus,

**VT** ventral tube,

**d0** unpaired chaeta on area frontalis of the head,

**p0** unpaired chaeta on abdominal terga IV and VI.

## Taxonomy

### Deuteraphorura pieroluccii sp. n.

Taxon classificationAnimaliaCollembolaOnychiuridae

http://zoobank.org/A8250415-1C64-4F4D-A120-EC8F2DE9913F

**Figure 1.**
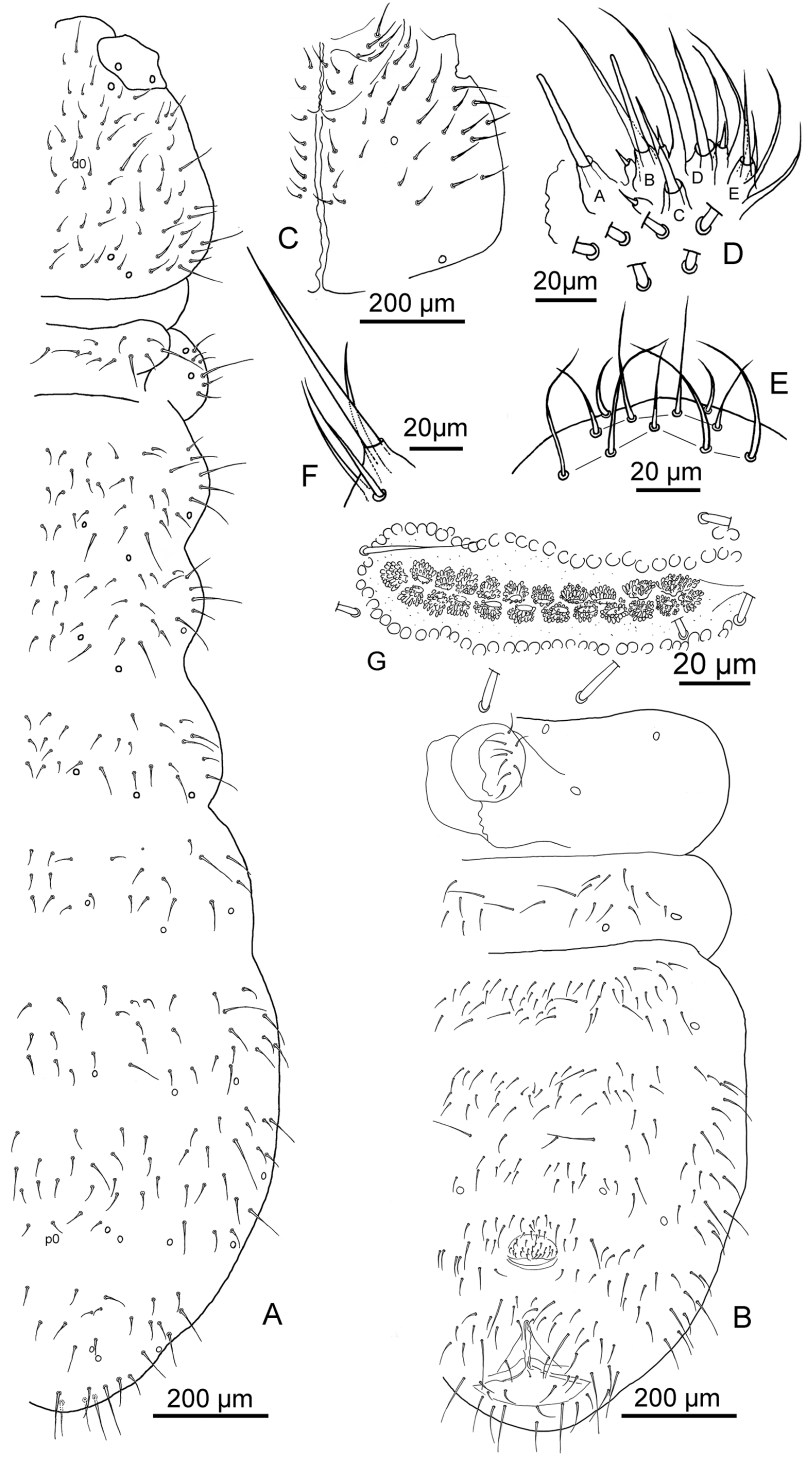
*Deuteraphorura pieroluccii* sp. n. **A** dorsal chaetotaxy **B** ventral chaetotaxy of the abdomen **C** ventral chaetotaxy of the head **D** chaetotaxy of the labial palp **E** chaetotaxy of the labrum **F** maxillary outer lobe **G** PAO.

**Figure 2.**
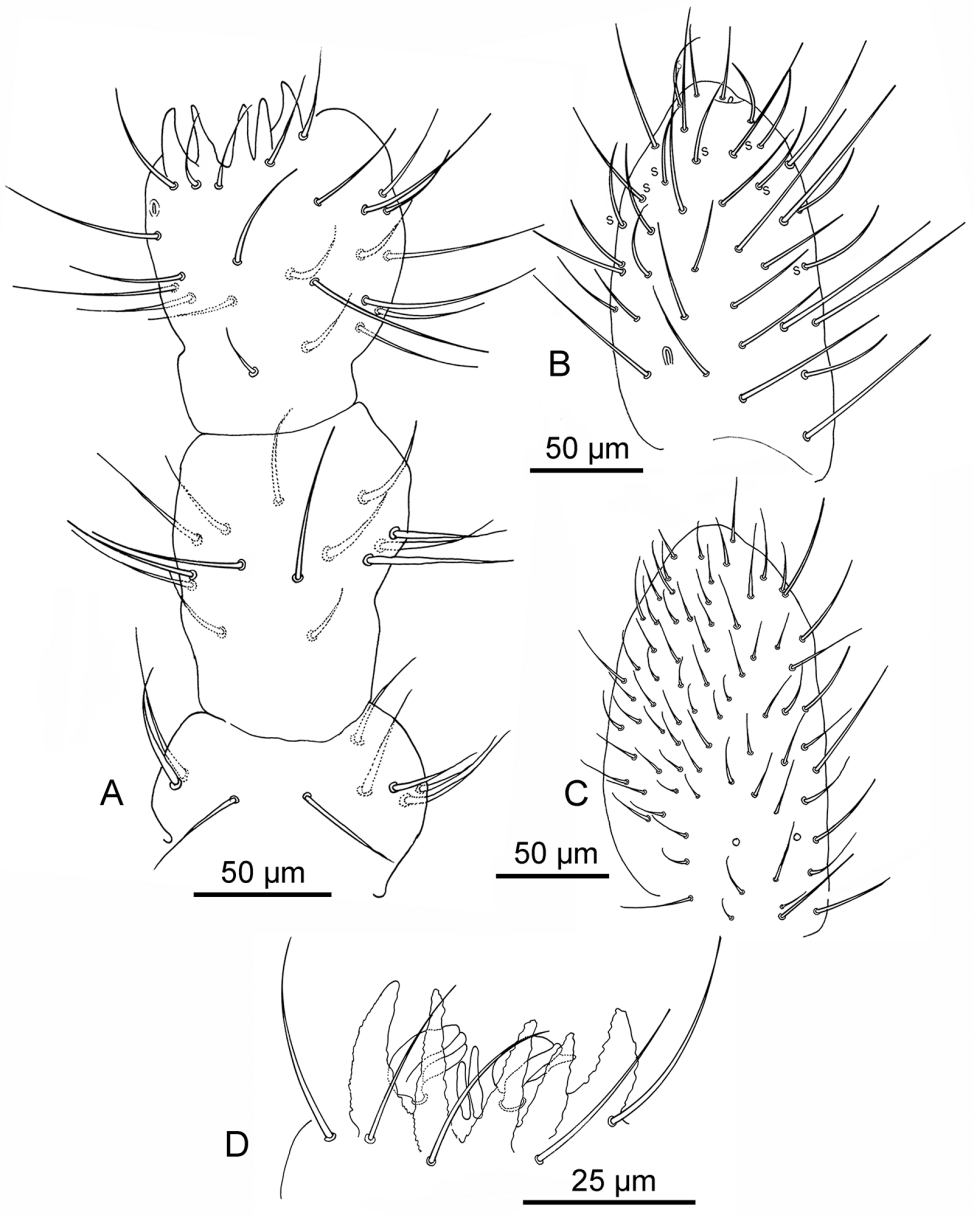
*Deuteraphorura pieroluccii* sp. n. **A** chaetotaxy of Ant. I-III **B** dorsal chaetotaxy of Ant. IV (s – sensillum) **C** ventral chaetotaxy of Ant. IV **D** antennal organ III.

**Figure 3.**
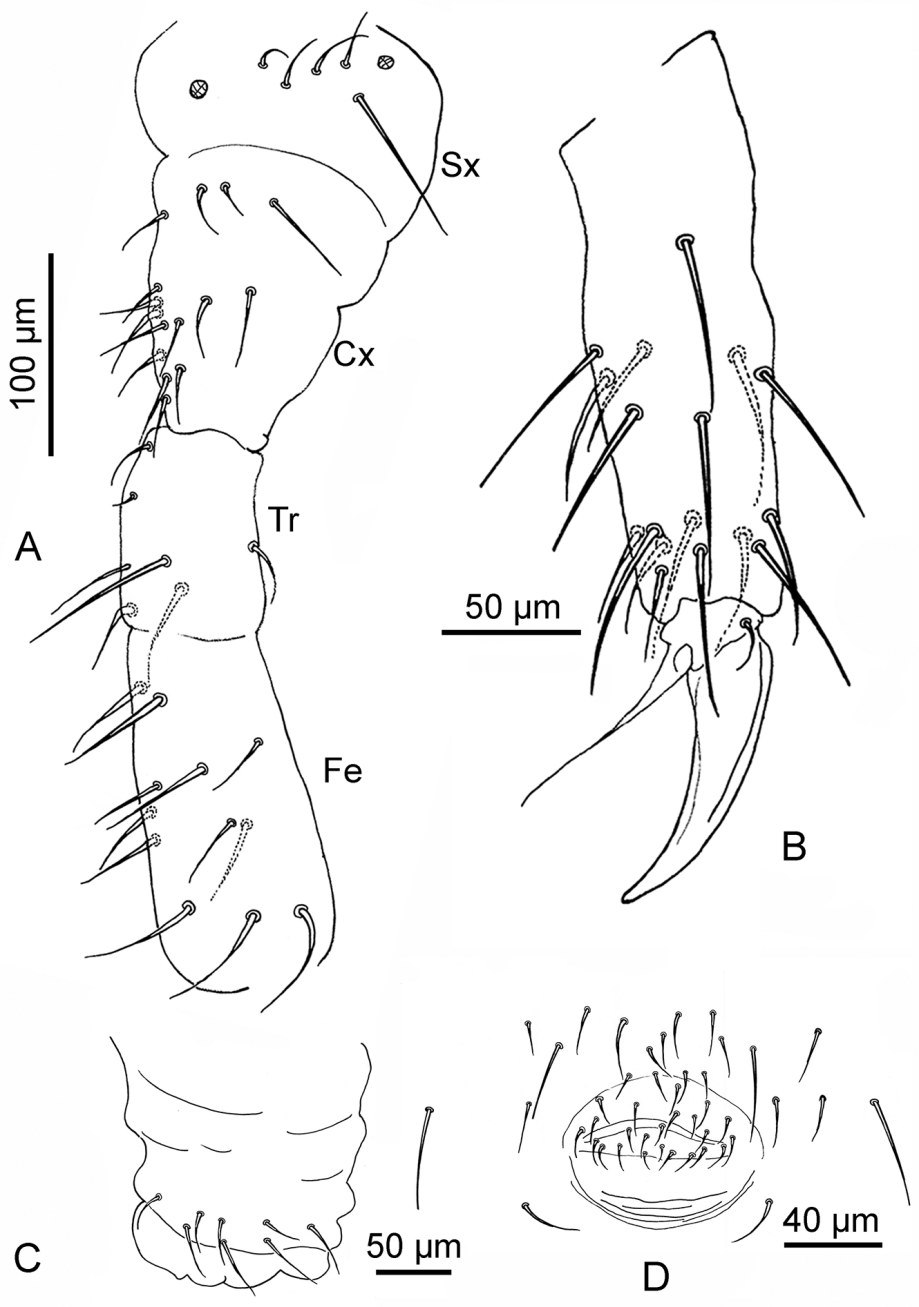
*Deuteraphorura pieroluccii* sp. n. **A** chaetotaxy of leg III from subcoxae to femur (Sx – subcoxae; Cx – coxae; Tr – trochanter; Fe – femur) **B** tibiotarsus III **C** ventral tube **D** female genital opening.

Figs 1 , 2 , 3 , Tables 1 , 2 , 3 , 4

#### Material examined.

Holotype female, Italy, Ravenna Province, near the locality Brisighella, Rontana Mount, 426 m asl, “Abisso Luigi Fantini” Cave (cave code ER RA 121; 44°13'23.08"N, 11°44'31.84"E), hand collected, 15 October 2013, leg. Poletti, Fabbri & Turri.

Paratypes: Abisso Luigi Fantini Cave, 1 female hand collected, 5 December 2013; Ravenna Province, Brisighella, locality of Monticino, 233 m asl, “Buco del Noce” Cave (cave code ER RA 107; 44°13'34. 68"N, 11°45'39.61"E), 4 females hand collected, 18 September 2013, leg. Poletti, Fabbri & Turri.

Holotype and five paratypes (all females) are deposited in the collembolan collection of the Department of Life Sciences, University of Siena.

#### Diagnosis.

Mean body length 2.7 mm. Pso formula dorsally: 32/033/33353; ventrally: 3/011/3212. Subcoxae I-III with 2 pso. AOIII made of five papillae, five guard chaetae, two small rods, two ribbed sensory clubs, and 1 ms. PAO with 21–22 compound vesicles, unpaired d0 chaeta present on the head. Abd. IV and VI with unpaired p0 chaeta. Without anal spines.

#### Description.

Average length 2.7 mm (2.4–3.1mm), body shape cylindrical with fine and uniform cuticle granulation. Colour in alcohol white. Length of Ants I, II, III and IV 65 μm, 120 μm, 130 μm and 170 μm, respectively. Antennae shorter than head (Ant/head diagonal ratio = 0.8). Area antennalis clearly -marked. Ant. IV with sub-apical organite and one ms at its base in ventro-lateral position. Sensilla on Ant. IV not clearly distinguishable from ordinary chaetae (Fig. 2B). Ant. I, II and III with 8–9, 13–14, and 18–19 chaetae, respectively (Fig. 2A). AOIII consisting of two ribbed sensory organs, two sensory rods, five papillae and five guard chaetae (Fig. 2D). Ms on Ant. III in ventro-lateral position below the level of the last guard chaeta of AOIII (Fig. 2A). PAO consists of 21–22 compound vesicles arranged in two parallel rows (Fig. 1G). Labrum with 5/4/2 chaetae as in Fig. 1C; labium (submentum) with 4 + 4 chaetae (Fig. 1E), basolateral field (mentum) with five chaetae; maxillary outer lobe with one basal chaeta and two sub-lobal hairs (Fig. 1F). Labial palp of AB type according to Fjellberg (1999) with six proximal chaetae; labial papillae A, B, C, D, and E with 1, 4, 0, 3, and 3 chaetae respectively (Fig. 1D), 6+6 postlabial setae along the ventral groove. Mandibles with strong molar plate and four apical teeth, jaws not clearly distinguishable in the preparations. VT with 8–9 + 8–9 abdominal lateral chaetae, without basal chaeta (Fig. 3D). Body chaetae differentiated into meso- and macrochaetae; Th.II – III with lateral ms. Dorsal chaetotaxy as in Fig. 1A, Tables 1–3. Dorsal cephalic chaeta d0 present (Fig. 1A). Abd. IV and V with p0 chaeta; anal spines absent. Extra chaetae and both left and right asymmetries have been observed. M/s ratio = 3.57 on Adb. V. Thoracic sterna without ventral chaetae. Ventral chaetotaxy of head and abdomen as in Fig. 1C, B. Furca reduced to a small papilla with 2 + 2 chaetae. Female genital plate with 25–27 chaetae (Fig. 3D). Tibiotarsi I, II and III with 18, 18, and 17 chaetae respectively; distal whorl with nine chaetae (Fig. 3B). Claw not elongated, without inner tooth; slender empodial appendage, without inner basal lamella, reaching 9/10 of the inner edge of the claw (Fig. 3B). Each subcoxa, coxa, trochanter, and femur with 5, 14–15, 7–9, and 12–15 chaetae respectively (Fig. 3A). Pso formula dorsally: 32/033/33353; ventrally: 3/011/3212; Subcoxae with two pso each. Parapseudocelli on sterna and femora weakly visible.

**Table 1.** *Deuteraphorura pieroluccii* sp. n. Dorsal chaetotaxy of the head. Abbreviations: m micro-mesochaetae; M macrochaetae.

row	
d	m0, m1, m2, m3, m4
sd	m1, m2, m3, m3’, m4, M5
sd’	m1, M2, m3, m4
v	m1, m2, m3, M4
ca	M5
cm	m3, m4
cb	m1, m2, m5, m6
cp	m1, m4, m6
p	m1, m2, m3, m4, m5
g	approx. 10 chaetae.

**Table 2.** *Deuteraphorura pieroluccii* sp. n. Dorsal chaetotaxy of the thorax. Abbreviations: m micro-mesochaetae; M macrochaetae;* – sometimes absent; ⚫ – variable in length.

row	Thorax I	Thorax II	Thorax III
a	m2, m4, m6	m2, m4, m5, m6, M7	m2, m3, m4, m5, m6, M7
m		m1, m2, m3*, m4 (m4’), M6⚫, M7	m1, m2, m3, m4, M6⚫, M7⚫
ca		m5, m6, M7	m5, M7
cp		m1, m2, m4, m6, M7	m1, m2, m4, m5, M7
p	M1, m2, m3, m4, M5, m6, M7	m1, m2, M3, m4, M5, m6, m7	m1, m2, M3, m4, M5, m6 (m6’), M7

**Table 3.** *Deuteraphorura pieroluccii* sp. n. Dorsal chaetotaxy of the abdomen. Abbreviations: m micro-mesochaetae; M macrochaetae; * – sometimes absent.

row	Abdomen I	Abdomen II	Abdomen III
a	m1, m2, m5, m6, m7	m1, m2, m4, m5*, m6, m7	m3, m5, m7
m	m1, m2, m3, M7	m1, m2*, m4, M7	m1, m2, m4, m5, M7, M8
ca	m4, m5, M7	m5, M7	m1, m4, M7, m8
p	m1, m2, m3, M4, m5, m6, M7	m1, m2, m3, M4, m5, M6, M7	m1, m2, m3, M4, m4’, m5, m6, M7, m8
**row**	**Abdomen IV**	**Abdomen V**	**Abdomen VI**
a	m1, m3, m4, m5, m6, m7, M8		
m	m1, M2, m3, m4, m5, m6, M7, m8	m2, m3, m5, m6, M7	
ca	m1, m2, m3, m4, m5, m6, m7	m2, m3, m4, m5	
p	m0, m1, m3, M5, m6, m7, M8	m1, m2, m3s, M5, m6, M7	M0, m1, M2, m3, m4

#### Etymology.

The species name is derived from Piero Lucci, former President of the Speleological Federation of Emilia-Romagna Region and current President of Speleo GAM Mezzano Caving Club. He was the coordinator of the speleological research project framework in which the new species was found.

#### Ecology.

The new species does not show particular morphological adaptations to the cave life; the claws, as well as the legs, the antennae and their sensillae are of normal shape, not elongated as is usually observed in the troglobitic species. However, to define whether or not a species is a true troglobitic species many other aspects of their biology and ecophysiology should be considered (Thibaud 1986), which at the moment cannot be ascertained. Specimens were collected in the two caves on winter bat guano in the internal part of the cave (aphotic with stable conditions). The quantity of guano in the caves is significant and is laid from more than one species of Chiroptera. *Deuteraphorura pieroluccii* sp. n. is to be considered as a guanobic element. The type locality of the new species is approximately 135 km from the type locality of *D. frasassii* (Fanciulli, 1999) (Frasassi Caves, Ancona, Italy).

#### Discussion.

The new species belongs to the group of *Deuteraphorura* without pseudocelli on the Th. I tergum. This character is typical of several *Deuteraphorura* species included in the updated identification key proposed by Weiner and Fiera (2014). Six species possess the same dorsal pso formula as *Deuteraphorura pieroluccii* sp. n. (32/033/33353): *D. bosnaria* (Gisin, 1964), *D. frasassii* (Fanciulli, 1999), *D. ossaria* (Gisin, 1964), *D. scotaria* (Gisin, 1954), *D. silesiaca* (Dunger, 1977), and *D. dianae* Weiner & Fiera, 2014. However, five of them differ from the new species by their ventral pso formulae: *D. bosnaria* 3/022/3212; *D. ossaria* 3/022/3222; *S. scotaria* 2/022/2212; *D. silesiaca* 2/011/1212; *D. dianae* 3/011/3112. *Deuteraphorura caprelleana* Fanciulli et al., 2010 and *D. pseudobosnaria* (Dallai, 1970) are similar to new species in their ventral pso formulae (3/011/3212), but differ in the dorsal pso formulae (*D. caprelleana*: 32/033/33354; *D. pseudobosnaria*: 33/033/33353). The dorsal and ventral pso formulae of the new species correspond to *D. frasassii* (Fanciulli, 1999) which may be distinguished by body size (2.6–3.1 mm in *Deuteraphorura pieroluccii* sp. n. vs. 1.3–1.6 mm in *D. frasassii*), number of distal chaetae on ventral tube (8–9 + 8–9 in *Deuteraphorura pieroluccii* sp. n. vs. 6–7 + 6–7 in *D. frasassii*), number of postlabial setae along the ventral groove (6+6 in *Deuteraphorura pieroluccii* sp. n. vs 4+4 in *D. frasassii*), and the number of compound vesicles in PAO (21–22 in the new species vs. 16–17 in *D. frasassii*). Further differences, especially in the dorsal chaetotaxy, between the two species are summarised in Table 4.

**Table 4.** Diagnostic characters between *D. pieroluccii* sp. n *and D. frasassii*.

Species	Body lenght	PAO compound vesicles	Setae tibiotarsi I-III	Postlabial setae ventral groove	Coxae	Ventral tube	Th. I
*D. frasassii*	1.6 mm	16–17	17,18,18	4+4	10–12	6–7	p3, p6 absent
*D. pieroluccii* sp. n.	2,7 mm	21–22	18,18,17	6+6	14–15	8–9	p3, p6 present
**Species**	**Th. II**	**Th. III**	**Abd. I**	**Abd. II**	**Abd. III**	**Abd. IV**	**Abd. V**
*D. frasassii*	m3 absent	m3 absent	m3 absent	a4 absent	m5, ca1 absent	a4, ca1, ca2 absent. p2 present	m3, ca2, ca3 absent
*D. pieroluccii* sp. n.	m3 sometimes absent	m3 present	m3 present	a4 present	m5, ca1 present	a4, ca1, ca2 present. p2 absent	m3, ca2, ca3 present

### Key to the Italian species of *Deuteraphorura* (based on dorsal and ventral formulae of pseudocelli; modified from Fanciulli et al. 2010)

**Table:** 

1	Th. I without pso	**2**
–	Th. I with 1+1 pso	**10**
2	Hind margin of the head with 3+3 pso	**3**
–	Hind margin of the head with 2+2 pso	**4**
3	Abd. I–IV ventrally with 3212, Abd. IV with chaeta p4	***D. pseudobosnaria* (Dallai)**
–	Abd. I–IV ventrally with 1212, Abd. tergum IV without chaeta p4	***D. apuanica* (Dallai)**
4	Abd. I with 5+5	***D. spipolae* (Massera)**
–	Abd. I with 3+3 pso	**5**
5	Abd. V with 3+3 pso	**6**
–	Abd. V with 4+4 pso	**8**
6	Abd. I–IV ventrally with 2212, PAO with 18–20 compound vesicles, body length 1.8–2.5 mm	***D. banii* Dallai & Fanciulli**
–	Abd. I–IV ventrally with 3212	**7**
7	PAO with 16–17 compound vesicles, body length 1.3–1.4 mm	***D. frasassii* (Fanciulli)**
–	PAO with 21–22 compound vesicles, body length 2.6–3.1 mm	***D. pieroluccii* sp. n.**
8	Head ventrally with 2+2 pso	***D. ghidinii* (Denis)**
–	Head ventrally with 3+3 pso	**9**
9	Abd. I–IV ventrally with 3212, body length 2,4–2,6 mm, PAO with 19–21 compound vesicles, Abd. IV without Ca0 chaeta	***D. caprelleana* Fanciulli, Loreti & Dallai**
–	Abd. I–IV ventrally with 2212, body length 1,41–1,85 mm, PAO with 14–16 compound vesicles, Abd. IV with Ca0 chaeta	***D. pseudoghidinii* (Dallai)**
10	Abd. V ventrally with 3+3 pso	**11**
–	Abd. V ventrally with 4+4 pso	**13**
11	Head ventrally with 2+2 pso	***D. eduardi* (Denis)**
–	Head ventrally with 3+3 pso	**12**
12	Abd. I–IV ventrally with 3222	***D. silvaria* (Gisin)**
–	Abd. I–IV ventrally with 1212	***D. pseudoinsubraria* (Dallai)**
13	Head ventrally with 2+2 pso	**14**
–	Head ventrally with 3+3 pso	**15**
14	Pso ventrally as 2/000/2212, PAO with 20 compound vesicles	***D. bergamaria* (Gisin)**
–	Pso ventrally as 2/011/1212, PAO with 15 compound vesicles	***D. defensaria* (Gisin)**
15	Pso ventrally as 3/011/2212	**16**
–	Pso ventrally as 3/011/3212	***D. cebennaria* (Gisin)**
16	Posterior part of Abd. V with only one macrochaeta positioned laterally to the most lateral pso	***D. imperfecta* (Denis)**
–	Posterior part of Abd. V with two macrochaetae, one between pso a, other laterally to pso d	***D. dunaria* (Gisin)**

## Supplementary Material

XML Treatment for Deuteraphorura pieroluccii
